# Prospective evaluation of whole-body MRI and ^18^F-FDG PET/MRI in N and M staging of primary breast cancer patients

**DOI:** 10.1007/s00259-020-04801-2

**Published:** 2020-04-24

**Authors:** Nils Martin Bruckmann, Lino M. Sawicki, Julian Kirchner, Ole Martin, Lale Umutlu, Ken Herrmann, Wolfgang Fendler, Ann-Kathrin Bittner, Oliver Hoffmann, Svjetlana Mohrmann, Frederic Dietzel, Marc Ingenwerth, Benedikt M. Schaarschmidt, Yan Li, Bernd Kowall, Andreas Stang, Gerald Antoch, Christian Buchbender

**Affiliations:** 1 Medical Faculty, Department of Diagnostic and Interventional Radiology, University Dusseldorf, Dusseldorf, Germany; 2 Department of Diagnostic and Interventional Radiology and Neuroradiology, University Hospital Essen, University of Duisburg-Essen, Essen, Germany; 3 Department of Nuclear Medicine, University Hospital Essen, University of Duisburg-Essen, Essen, Germany; 4 Department Gynecology and Obstetrics, University Hospital Essen, University of Duisburg-Essen, Essen, Germany; 5 Department of Gynecology, Medical Faculty, University Dusseldorf, Dusseldorf, Germany; 6 Institute of Pathology, University Hospital Essen, West German Cancer Center, University Duisburg-Essen and the German Cancer Consortium (DKTK), Essen, Germany; 7 Institute of Medical Informatics, Biometry and Epidemiology, University Hospital of Essen, Essen, Germany

**Keywords:** PET/MRI, MRI, Breast cancer staging

## Abstract

**Objectives:**

To evaluate and compare the diagnostic potential of whole-body MRI and whole-body ^18^F-FDG PET/MRI for N and M staging in newly diagnosed, histopathologically proven breast cancer.

**Material and methods:**

A total of 104 patients (age 53.4 ± 12.5) with newly diagnosed, histopathologically proven breast cancer were enrolled in this study prospectively. All patients underwent a whole-body ^18^F-FDG PET/MRI. MRI and ^18^F-FDG PET/MRI datasets were evaluated separately regarding lesion count, lesion localization, and lesion characterization (malignant/benign) as well as the diagnostic confidence (5-point ordinal scale, 1–5). The N and M stages were assessed according to the eighth edition of the American Joint Committee on Cancer staging manual in MRI datasets alone and in ^18^F-FDG PET/MRI datasets, respectively. In the majority of lesions histopathology served as the reference standard. The remaining lesions were followed-up by imaging and clinical examination. Separately for nodal-positive and nodal-negative women, a McNemar chi^2^ test was performed to compare sensitivity and specificity of the N and M stages between ^18^F-FDG PET/MRI and MRI. Differences in diagnostic confidence scores were assessed by Wilcoxon signed rank test.

**Results:**

MRI determined the N stage correctly in 78 of 104 (75%) patients with a sensitivity of 62.3% (95% CI: 0.48–0.75), a specificity of 88.2% (95% CI: 0.76–0.96), a PPV (positive predictive value) of 84.6% % (95% CI: 69.5–0.94), and a NPV (negative predictive value) of 69.2% (95% CI: 0.57–0.8). Corresponding results for ^18^F-FDG PET/MRI were 87/104 (83.7%), 75.5% (95% CI: 0.62–0.86), 92.2% (0.81–0.98), 90% (0.78–0.97), and 78.3% (0.66–0.88), showing a significantly better sensitivity of ^18^F-FDG PET/MRI determining malignant lymph nodes (*p* = 0.008). The M stage was identified correctly in MRI and ^18^F-FDG PET/MRI in 100 of 104 patients (96.2%). Both modalities correctly staged all 7 patients with distant metastases, leading to false-positive findings in 4 patients in each modality (3.8%). In a lesion-based analysis, ^18^F-FDG PET/MRI showed a significantly better performance in correctly determining malignant lesions (85.8% vs. 67.1%, difference 18.7% (95% CI: 0.13–0.26), *p* < 0.0001) and offered a superior diagnostic confidence compared with MRI alone (4.1 ± 0.7 vs. 3.4 ± 0.7, *p* < 0.0001).

**Conclusion:**

^18^F-FDG PET/MRI has a better diagnostic accuracy for N staging in primary breast cancer patients and provides a significantly higher diagnostic confidence in lesion characterization than MRI alone. But both modalities bear the risk to overestimate the M stage.

## Introduction

Breast cancer is the most common cancer in women worldwide with approximately 2.1 million new cases every year [1]. As in most malignancies, breast cancer mortality increases with the individual tumor burden, while management and prognosis depend heavily on the initial tumor stage [2]. Therefore, for optimal treatment and better survival, precise initial staging plays a pivotal role. Herein, the correct determination of the lymph node status and the detection of distant metastases are of utmost importance. Treatment of breast cancer patients without distant metastases usually includes surgery and chemotherapy, alongside irradiation or further drug therapy before and after surgery [3]. Depending on primary tumor size and locoregional metastases, the surgical procedure of choice can go from breast-preserving resection to complete mastectomy and dissection of the ipsilateral axillary and subclavian lymph nodes. In patients with proven distant metastases a palliative concept is intended, including extensive systemic therapy [4].

The current diagnostic algorithm comprises plain mammography, ultrasound, and in some cases magnetic resonance imaging (MRI) of the breast to evaluate the local tumor extent [3, 5]. Especially the demand for dedicated breast MRI has heavily increased over the last few years [6]. Due to a growing understanding of the importance of an accurate initial staging of breast cancer patients, whole-body imaging with computed tomography (CT) has recently been established in addition to bone scintigraphy for the detection of locoregional and distant metastases [3, 7]. However, whole-body MRI is rarely used for initial staging of breast cancer [8], despite the option of combining dedicated breast MRI with a whole-body examination and its well-known advantages when imaging parenchymal organs [9, 10]. When it comes to PET recent studies have reported a high diagnostic accuracy of ^18^F-fluorodeoxyglucose-positron emission tomography/CT (^18^F-FDG PET/CT) in distant breast cancer metastases [11–13]. Consequently, hybrid ^18^F-FDG PET/MRI might serve as a comprehensive “all-in-one” breast cancer staging tool, providing precise local and whole-body staging in one procedure. In smaller cohorts, ^18^F-FDG PET/MRI has already shown promising results as an alternative modality in primary breast cancer staging [14–18] and in recurrent disease [19–22].

Therefore, the purpose of this prospective study was to evaluate the diagnostic accuracy of whole-body MRI compared with whole-body ^18^F-FDG PET/MRI for the initial N and M staging in a large cohort of therapy-naive breast cancer patients.

## Material and methods

### Patients

This prospective, multi-center study was approved by the institutional review board of the University Duisburg-Essen (study number 17-7396-BO) and Düsseldorf (study number 6040R), and all patients signed a written informed consent form prior to enrolment. Between August 2017 and June 2019, a total of 104 female patients (53.4 ± 12.5, range 29–84 years, Table 1) with newly diagnosed breast cancer were included if they met the following inclusion criteria: [1] Newly diagnosed, treatment-naive T2-tumor or higher T-stage or [2] newly diagnosed, treatment-naive triple-negative tumor of every size or [3] newly diagnosed, treatment-naive tumor with molecular high risk (T1c, Ki67 > 14%, HER2-new over-expression, G3). Exclusion criteria were former malignancies in the last 5 years, contraindications to MRI or MRI contrast agents and pregnancy or breast-feeding. All enrolled patients underwent ^18^F- FDG PET/MRI.

**Table 1:** Patients demographics

		*N* (%)
Total patients		104(100)
Menopause status	Pre	43
	Peri	11
	Post	50
Family risk profile	Positive	11
	Negative	93
BRCA-1	Positive	1
	Negative	27
	Unknown	76
BRCA-2	Positive	2
	Negative	26
	Unknown	76
Ki 67	Positive (> 14%)	88
	Negative (< 14%)	16
PR status	Positive	74
	Negative	30
ER status	Positive	77
	Negative	27
HER2-neu expression	0	42
	1+	33
	2+	11
	3+	18
Subtype	Luminal a	12
	Luminal b	74
	HER2-enriched	2
	Basal-like	16
Tumor Grade	G1	2
	G2	60
	G3	42
Histology	Ductal invasive/NST	97
	Lobular invasive	5
	Mucinous invasive	1
	Mixed type	1

### PET/MRI

The ^18^F-FDG PET/MRI examinations were performed on an integrated 3.0-Tesla Biograph mMR scanner (Siemens Healthcare GmbH, Erlangen, Germany). To ensure blood glucose levels below 150 mg/dl, blood samples were obtained prior to the injection of a body-weight adapted dose of ^18^F-FDG (4 MBq/kg bodyweight), resulting in a mean activity of 253.8 ± 42.6 MBq. All patients underwent whole-body ^18^F-FDG PET/MRI in supine position from head to the mid-thigh using a dedicated 16-channel head-and neck radiofrequency (RF) coil, a 24-channel spine-array RF coil and referring to the patients height three to five flexible 6-channel body array RF coils. PET images were performed simultaneously with the MRI data acquisition and with an acquisition time of 3 min per bed position in four or five positions, depending on the patients’ height (axial FOV 25.8 cm, matrix size 344 × 344). Mean duration time according to manufacturer’s specifications is set at 40 min for the whole-body examination. PET data sets were reconstructed utilizing an iterative ordered-subset expectation maximization (OSEM) algorithm with three iterations and 21 subsets.

For MR-based PET attenuation correction, a two-point (fat, water) coronal 3D-Dixon-VIBE sequence was acquired to generate a four-compartment model (background air, lungs, fat, muscle).

The dedicated ^18^F- FDG PET/MRI protocol consisted of the following sequences:

1. A transverse T2–weighted (T2w) fat-suppressed half Fourier acquisition single shot turbo spin echo (HASTE) sequence in respiratory medium position and a slice thickness of 7 mm.
2. A transverse diffusion-weighted echo-planar imaging (EPI DWI) sequence (*b* values 0, 500, 1000) in respiratory medium position with a slice thickness of 5 mm.
3. A transversal T1–weighted (T1w) fat saturated post-contrast volume-interpolated breath-hold examination (VIBE) sequence after intravenous injection of a gadolinium-based contrast agent (0.2 mmol/kg body weight, Dotarem, Guerbet GmbH, Germany) with a slice thickness of 3 mm.

As part of the ^18^F- FDG PET/MRI examination, a dedicated breast PET/MRI in head-first prone position was performed in all patients prior to whole-body imaging. The presented analysis is based on data of a larger prospective study. Therefore, in consideration of the focus of the presented study, these dedicated breast MRI sequences were not included in evaluation.

### Image analysis

MRI and ^18^F-FDG PET/MRI images were analyzed separately by two experienced radiologists in hybrid imaging and MR imaging with a reading gap of at least 4 weeks to avoid recognition bias. The datasets were evaluated on a dedicated OsiriX workstation (Osirix MD v.9.0.2, Pixmeo, SARL, Bernex, Switzerland). The readers were aware of the diagnosis but blinded to results of N and M stages and results from prior imaging (e.g., sonography). For every patient, the number of lesions, the lesion type (malignant / benign), location, and size as well as the diagnostic confidence of lesion type ratings (5-point ordinal scale, 1 = very low confidence, 2 = low confidence, 3 = indeterminate confidence, 4 = high confidence, 5 = very high confidence) were determined in MRI alone and ^18^F-FDG PET/MRI. Discrepant interpretations were resolved by consensus decision-making in a separate session between the two readers. Lymph nodes were classified as malignant based on morphological and metabolic criteria, comprising short-axis diameter > 10 mm, spherical configuration, shape (smooth vs. irregular), increased contrast enhancement, diffusion restriction, and focally increased FDG uptake [22, 23]. In accordance with previous publications, findings were considered to be malignant for the evaluation of distant metastases when showing an invasive growth pattern, central necrosis, and typically malignant MR signal characteristics like pathological contrast enhancement and diffusion restriction. On ^18^F-FDG PET/MRI a visually detectable focal uptake of FDG above background signal counted as a sign of malignancy. The standardized uptake value (SUVmax) was measured in every lesion with a focal FDG uptake by placing a manually drawn polygonal volume of interest (VOI) over each lesion on attenuation-corrected PET images. In all lesions, the maximum diameter was measured.

### Reference standard

The 104 patients enrolled in this study had a total of 298 lesions, excluding the primary tumor mass. In 98 patients, 204 out of 298 lesions were confirmed histopathologically. A surrogate reference standard was applied to the remaining 94 lesions containing follow-up imaging and clinical examinations. A decrease in size of suspicious lesions after therapy was regarded as a sign of malignancy. Forty-five lesions were followed-up by CT and 19 lesions by MRI (mean interval 8 ± 5 months). The remaining 30 lesions were followed-up with sonography and clinical examination.

### Statistical analysis

Statistical analysis was performed using SPSS Statistics 22 (IBM Inc., Armonk, NY, USA) and Graphpad Prism 7 (GraphPad Software, La Jolla, CA, USA). All data are presented as mean ± standard deviation. The data were analyzed calculating sensitivity, specificity, positive and negative predictive values (PPV, NPV) on a per-patient basis, and a per-lesion basis. Separately for nodal-positive and nodal-negative patients, a McNemar chi^2^ test was performed to compare sensitivity and specificity between MRI alone and ^18^F-FDG PET/MRI. We used a Wilcoxon signed rank test to compare the diagnostic confidence of lesion nature assessments (benign/malignant). A *p* value of less than 0.05 was set as indicating a statistical significance.

## Results

### Patient-based analysis

**Fig. 1:**
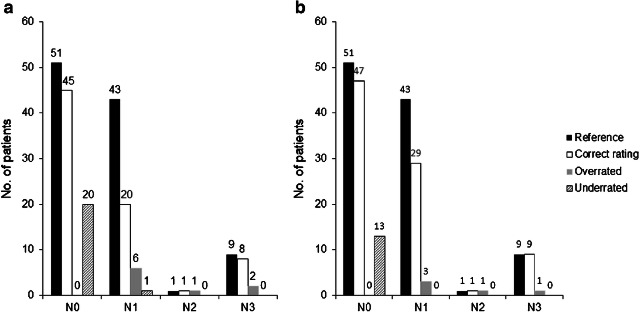
Determination of the lymph node stage with MRI alone (**a**) and ^18^F-FDG PET/MRI (**b**)

When differentiating between nodal-positive and nodal-negative patients, MRI rated 78/104 (75%, 95% CI 65.5–83.0) of the patients correctly, leading to a sensitivity of 62.3% (95% CI: 47.9–75.2), a specificity of 88.2% (95% CI 76.1–95.6), a PPV of 84.6% (95% CI 69.5–94.1), and a NPV of 69.2% (95% CI 56.6–80.1) (see Table 2). The exact N stage (i.e., N0, N1, N2, N3) was determined correctly by MRI in 74 of 104 patients (71.2%, 95% CI 61.5–79.6). The specific distribution of lymph nodes is shown in Fig. 1.

**Table 2:** N staging on a patient-based analysis. Distribution of N staging for MRI alone and ^18^F-FDG PET/MRI and comparison with the reference standard

	Standard of reference			
N stage MRI	Nodal negative	Nodal positive	Total	
Nodal negative	45	20	65	
Nodal positive	6	33	39	
Total	51	53		
Correct N ratings				*78* (*75.0%*)
N stage PET/MRI				
Nodal negative	47	13	60	
Nodal positive	4	40	44	
Total	51	53		
Correct N ratings				*87* (*83.7%*)

With ^18^F-FDG PET/MRI differentiation between nodal-positive and nodal-negative patients was rated correctly in 87/104 (83.7%, 95% CI 75.1–90.2) with a sensitivity of 75.5% (95% CI 61.7–86.2), a specificity of 92.2% (95% CI 81.1–97.8), a PPV 90.9% (95% CI 78.3–97.5), and a NPV of 78.3% (95% CI 65.8–87.9). The exact N stage was determined correctly in 86/104 (82.7%, 95% CI 74.0–89.4) of the patients (Fig. 1). A total of 20/53 (37.7%, 95% CI 24.8–52.1) nodal-positive patients were missed by MRI, while only 13/53 (24.5%, 95% CI 13.8–38.3) nodal-positive patients were missed with ^18^F-FDG PET/MRI (Fig. 4). There were 6 (11.8%, 95% CI 4.4–23.9) false-positive lymph node findings in MRI and 4 (7.8%, 95% CI 2.2–18.9) in PET/MRI.

For nodal-positive women, the exact McNemar chi^2^ test indicated that nodes were more often found by ^18^F-FDG PET/MRI than by MRI alone (test statistic = 7.0, *p* = 0.002). The corresponding difference in sensitivities was 13.2% (95% CI − 4.2–30.7). For nodal-negative women, the test statistic of the exact McNemar chi^2^ test was 2.0 (*p* = 0.50). The corresponding difference in specificities was 3.9% (95% CI − 7.6–15.4%). Table 2 gives a detailed overview of N stage performance with MRI and ^18^F-FDG PET/MRI.

**Fig. 2:**
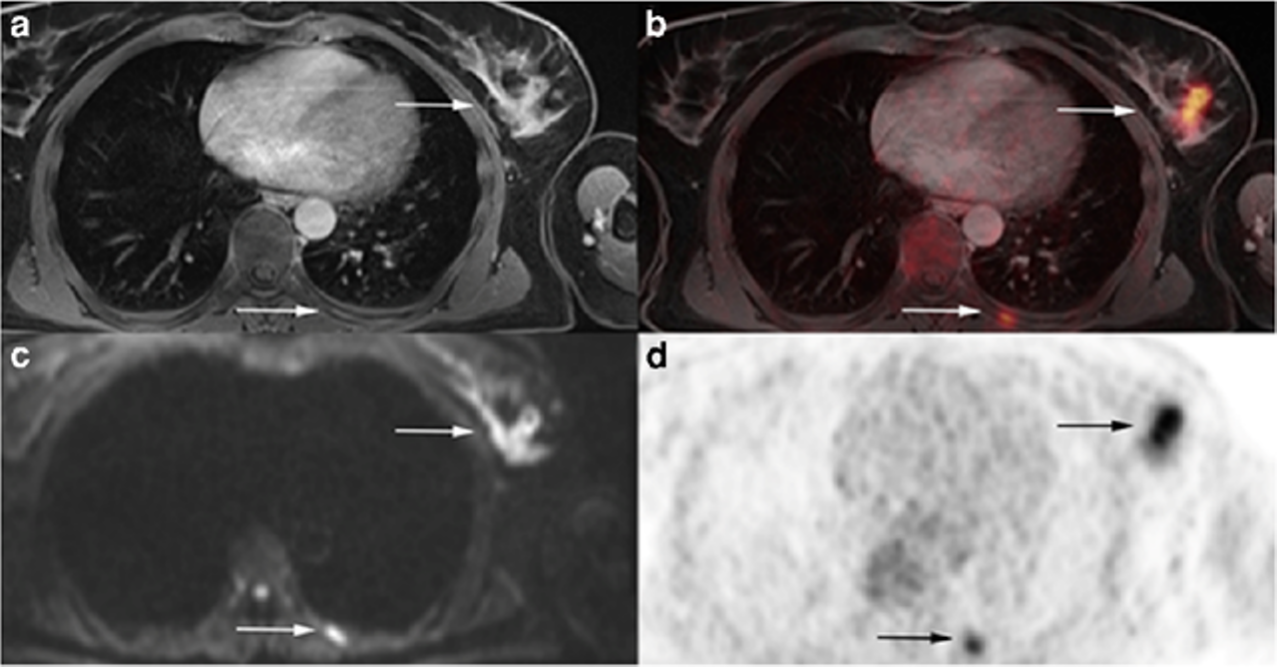
A 57-year old woman with diagnosis of primary breast cancer. Primary tumor located in the left breast and visible bone metastasis in a left rib with contrast enhancement on T1w fs VIBE (**a**), corresponding diffusion restriction (**c**), and pathological FDG uptake on PET (**d**) and fused ^18^F-FDG PET/MRI (**b**)

**Fig. 3:**
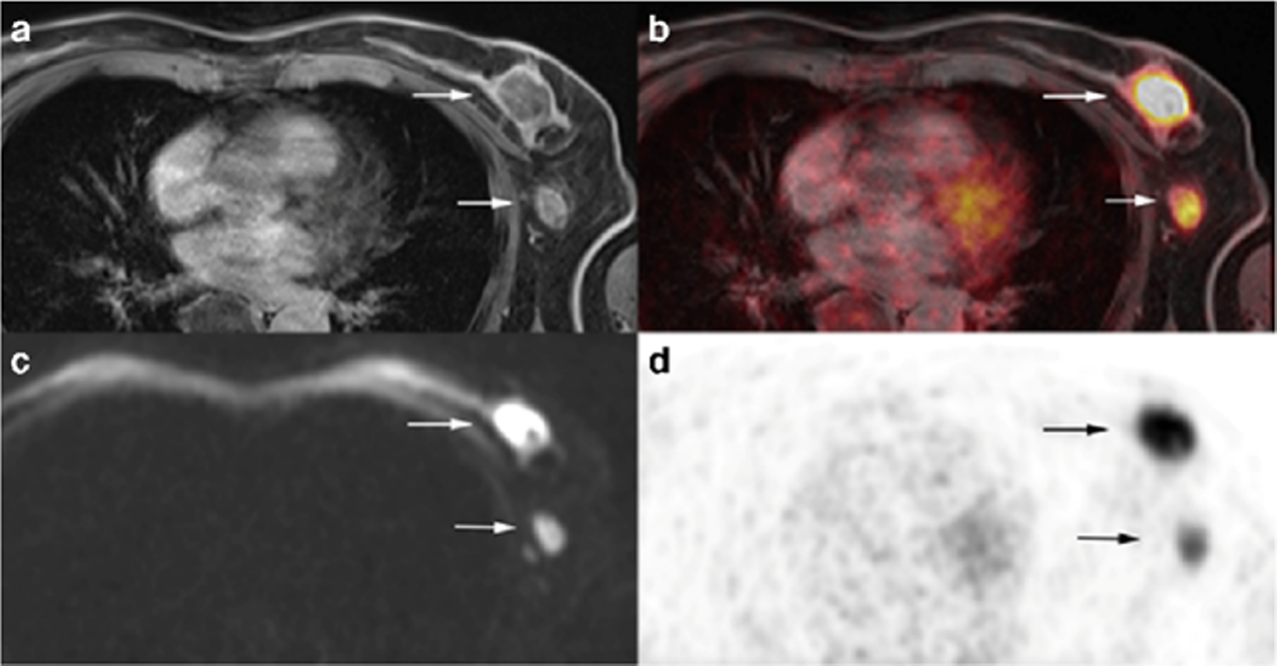
A 47-year old woman with primary breast cancer on the left side. Visible enlarged axillary lymph node with contrast enhancement in T1w fs VIBE (**a**) and corresponding diffusion restriction (**c**) as well as a pathological FDG uptake on PET (**d**) and fused ^18^F-FDG PET/MRI (**b**), rated as an axillary lymph node metastasis

**Fig. 4:**
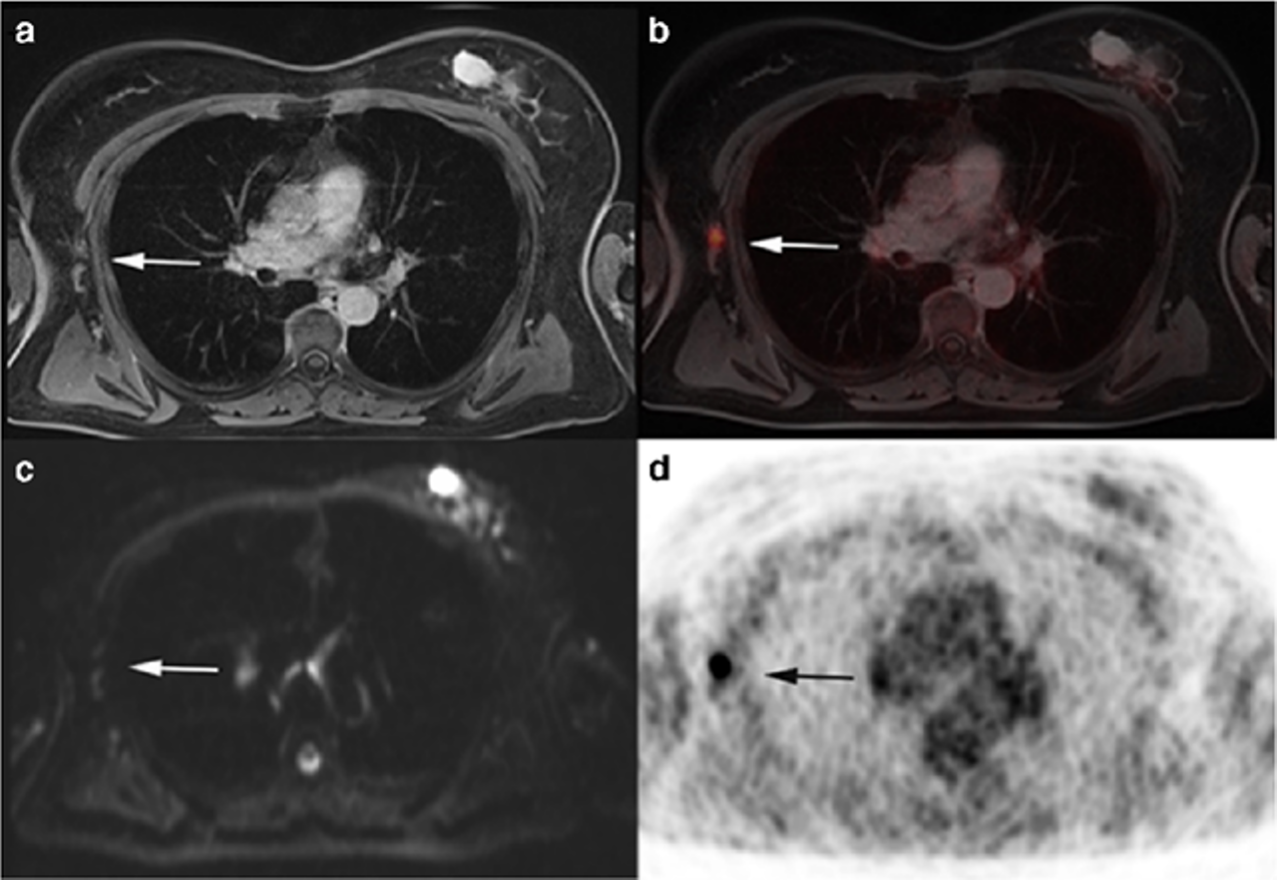
A 61-year old woman with diagnosis of primary breast cancer. Not enlarged, ovoid axillary lymph nodes in T1w fs VIBE without contrast enhancement and with visible fatty hilum (**a**). No evidence of a clear diffusion restriction (**c).** However, a pathological FDG uptake on PET (**d**) and fused ^18^F-FDG PET/MRI (**b**) is visible, indicating an axillary lymph node metastasis. Accordingly, histopathology confirmed malignancy

According to the reference standard distant metastases were present in 7/104 patients (6.7%, Table 3, Figs. 2 and 3). The M stage was defined correctly with MRI and ^18^F-FDG PET/MRI in 100 of 104 patients. As both modalities correctly detected all patients with proven distant metastases, there were false-positive findings in 4 patients (3.8%, 95% CI 1.1–9.6) in each modality, resulting in a sensitivity of 100% (95% CI 59.0–100.0), a specificity of 95.9% (95% CI 90.4–98.9), a NPV of 100% (95% CI 96.3–100.0), and a PPV of 63.7% (95% CI 30.8–89.1). Three of the false-positive ratings were identical in both modalities, comprising one patient with a focal pericarditis showing a normal follow-up MRI after 12 months, one patient with two suspicious lung lesions that were followed-up by CT after 2 months without any sign of malignancy and one patient with multiple enlarged abdominal lymph nodes, which turned out benign in a histopathological examination and on follow-up MRI after 1 year. Additionally, ^18^F-FDG PET/MRI identified one patient with a suspicious liver lesion and MRI determined a pararenal and a subcutaneous mass in another patient. Both these lesions were non-malignant according to follow-up imaging (Fig. 4).

**Table 3:** M staging on a patient-based analysis. Distribution of M staging for MRI and ^18^F-FDG PET/MRI and comparison to the reference standard. This table is identical for both modalities

	Standard of reference			
M stage MRI and PET/MRI	Negative	Positive	Total	
Negative	93	0	93	
Positive	4	7	11	
Total	97	7		
Correct N and M in MRI				*72* (*69.2%*)
Correct N and M in PET/MRI				*84* (*80.8%*)

### Lesion-based analysis

In accordance with the reference standard, a total of 298 lesions, containing 155 malignant (52%) and 143 benign lesions (48%), were included in the final analysis (see Table 4*,* Fig. 3). ^18^F-FDG PET/MRI showed a higher diagnostic accuracy in the lesion-based analysis than MRI alone with 258 vs. 224 correct lesion nature ratings (86.6% vs. 75.2%, difference: 11.4% (95% CI 5.1–17.7)). Furthermore, the McNemar chi^2^ test indicated a significant difference for correct malignant lesion rating between MRI alone and ^18^F-FDG PET/MRI (104 vs. 133 correct lesion nature ratings, 67.1% vs. 85.8%, difference 18.7% (95% CI 9.5–27.9), *p* < 0.0001) and an equivalent result in detecting benign lesions (120 vs. 125 correct lesion nature ratings, 83.9% vs. 87.4%, difference 3.5% (95% CI − 4.6–11.6), *p* = 0.063) (see Table 5). In detail, ^18^F-FDG PET/MRI had 22/155 (14.2%, 95% CI 9.1–20.7) false-negative ratings of axillary and subclavian lymph nodes, due to small lesion size and weak FDG uptake, while MRI alone misinterpreted a total of 38/155 (24.5%, 95% CI 18.0–32.1) of the malignant lesions as not malignant. ^18^F-FDG PET/MRI correctly identified all of the 31 distant metastases and did not miss any of the malignant lesions while MRI failed to detect 5 bone metastases in one patient and one malignant hilar lymph node as well as seven non-enlarged lymph node metastases in clavicular and mammarian position. Moreover, there were 19 and 18 histopathologically proven false-positive findings in MRI and ^18^F-FDG PET/MRI, respectively, due to elevated size, suspicious shape, or increased FDG uptake.

**Table 4:** Location of all 155 malignant lesions according to the standard of reference

	Location	Number	Percentage
Distant	Bone metastases	28	18.1
	Lung metastases	2	1.3
	Hilar lymph node	1	0.6
Locoregional	Lymph node metastases	124	80
	Axillary	102	
	Clavicular	12	
	Subpectoral	2	
	Cervical	1	
	Internal mammarian artery	7	
Total		155	100

**Table 5:** Lesion-based analysis. Correct ratings, false ratings and missed lesions on MRI alone and ^18^F-FDG PET/MRI in relation to the total number of malignant and benign according to the reference standard

		Malignant lesions	Benign lesions
MRI	Correct ratings	104 (67.1%)	120 (83.9%)
	False ratings	38 (24.5%)	19 (13.3%)
	Missed lesions	13 (8.4%)	4 (2.8%)
	Total	155 (100%)	143 (100%)
PET/MRI	Correct ratings	133 (85.8%)	125 (87.4%)
	False ratings	22 (14.2%)	18 (12.6%)
	Missed lesions	0 (0%)	0 (0%)
	Total	155 (100%)	143 (100%)

### Diagnostic confidence

^18^F-FDG PET/MRI showed a significantly higher overall diagnostic confidence than MRI alone (4.1 ± 0.7 vs. 3.4 ± 0.7, *p* < 0.0001). Comparing the diagnostic confidence regarding malignant lesions only, containing locoregional and distant metastatic lesions, ^18^F-FDG PET/MRI was also significantly superior to MRI alone (4.3 ± 0.7 vs. 3.4 ± 0.7, p < 0.0001). Comparing the diagnostic confidence regarding benign lesions only, significant differences in favor of ^18^F-FDG PET/MRI were observed (3.8 ± 0.7 vs. 3.3 ± 0.7, *p* < 0.001).

## Discussion

This study shows that both the whole-body ^18^F-FDG PET/MRI and whole-body MRI are valuable diagnostic tools for staging breast cancer patients. ^18^F-FDG PET/MRI outperforms the accuracy of MRI alone when assessing the N stage, and the diagnostic confidence is significantly higher with ^18^F-FDG PET/MRI.

Due to a growing understanding of the importance of an accurate initial staging, new staging modalities, primarily the CT, have been established and integrated into breast cancer guidelines [4, 7]. The demand for dedicated breast MRI has heavily increased over the last few years, and based on the growing usage of breast MRI, a subsequent implementation of a whole-body MRI is also conceivable.

In regard to the application of hybrid imaging modalities, the 2015 European Society For Medical Oncology (ESMO) and the 2016 National Comprehensive Cancer Network (NCCN) guidelines consider systemic staging with ^18^F-FDG PET/CT only for patients with inconclusive results in conventional imaging, in high-risk patients [7] or in patients with newly diagnosed stage III breast cancer, except for operable IIIA breast cancer [24]. However, recent studies showed that ^18^F-FDG PET/CT detects unsuspected distant metastases in up to 15% of patients compared to the traditional staging algorithm in patients with initial stage IIB breast cancer [13, 25, 26]. Since its introduction in 2011, there has been a large quantity of studies indicating a high diagnostic value of PET/MRI for whole-body cancer staging [27]. Several trials have already noted a superiority of PET/MRI compared with MRI alone in primary and recurrent cancer staging, for example in women with pelvic cancer [23, 28]. Furthermore, some initial studies showed similar results for the superiority of hybrid imaging modalities in detecting malignant lymph nodes and distant metastases in breast cancer [19, 29, 30].

Furthermore, it has been shown that ^18^F-FDG PET/MRI is superior to ^18^F-FDG PET/CT in the detection of breast cancer metastases [19]. This applies to axillary lymph node metastases, to liver and bone metastases, and to the total tumor stage [21, 31, 32]. It was emphasized in former studies that in combination with a dedicated breast PET/MRI protocol, ^18^F-FDG PET/MRI has the appealing potential of a one-stop-shop solution for patients with primary breast cancer [33, 34].The results of our study reveal a significantly better accuracy for determining the correct N stage with ^18^F-FDG PET/MRI than with MRI alone. Both modalities showed similarly strong results in specificity on a patient-based analysis for the N and M rating. The lesion-based analysis confirmed these results discovering a significant higher diagnostic accuracy of ^18^F-FDG PET/MRI especially in detecting malignant lesions with lower false-negative ratings, especially in malignant lymph nodes.

Regarding the detection of locoregional lymph node metastases, Grueneisen et al. described a higher sensitivity of PET-based imaging, comparing ^18^F-FDG PET/CT, ^18^F-FDG PET/MRI, and MRI alone in a study cohort of 49 primary breast cancer patients with sensitivities of 78%, 78%, and 67% and specificities of 94%, 90%, and 87%, supporting the results of our trial [32]. Ergul et al. also showed a higher performance of PET-based imaging for axillary metastases with a sensitivity of 67% and a specificity of 89% with ^18^F-FDG PET/CT, compared with 47% and 78% for MRI [35]. The sentinel lymph node biopsy is still the clinical standard for determining nodal-positive patients. In clinical routine, nodal-positive patients undergo axillary lymph node dissection (ALND) in a second surgical intervention. The traditional staging algorithm with clinical examination, sonography, conventional mammography, and breast MRI is a useful but still inadequate predictor of axillary lymph node involvement and is far away from serving as a potential alternative to invasive procedures [36, 37]. Thus, according to previous results and the results of our study, PET/MRI and PET/CT are imaging techniques with a more reliable selection of patients in nodal-positive and nodal-negative and could help to reduce surgical intervention, for example, identifying patients who should be treated with ALND immediately, avoiding a prior lymph node biopsy.

In our study, there was no difference between ^18^F-FDG PET/MRI and MRI alone when assessing the M stage. Both modalities were able to detect all of the seven patients with distant metastases but bear the risk of overestimating the M stage, rating four patients as false positive. In the study of Sawicki et al. [19], whole-body ^18^F-FDG PET/MRI reported superiority regarding detection of distant malignant lesions compared to whole-body MRI in recurrent breast cancer patients. Catalano et al. [38] compared whole-body ^18^F-FDG PET/MRI with whole-body DWI MRI in a smaller cohort study, yielding an insignificantly better performance of PET/MRI in predicting the initial whole-body tumor stage of breast cancer.

Other studies explored the expected high diagnostic potential of ^18^F-FDG PET/MRI and revealed a higher sensitivity of ^18^F-FDG PET/MRI over ^18^F-FDG PET/CT in the diagnosis of locoregional and distant metastases in breast cancer, especially regarding liver and bone metastases, the most common locations of distant breast cancer spread [19, 21, 39]. For instance, Catalano et al. [40] described a significantly higher identification of bone metastases in breast cancer by ^18^F-FDG PET/MRI compared with ^18^F-FDG PET/CT (141 vs. 90, *p* < 0.001) in 25 patients. In the study of Pace et al. [41], ^18^F-FDG PET/MRI showed equivalent performance to ^18^F-FDG PET/CT in terms of qualitative lesion detection. Only the overall detection and characterization of lung lesions remains inferior with ^18^F-FDG PET/CT, caused by the limited ability of MRI to detect small lung lesions [9, 10]. In view of our own results and results of the aforementioned previous studies, it can be summarized that regarding distant metastases detection ^18^F-FDG PET/MRI appears to have a high sensitivity but bears the risk of false-positive findings. From a clinical perspective, a final histopathological confirmation of suspicious lesions is still required.

Besides, the mere detection of potential lesions, in daily routine diagnostic or interpretation confidence, is also a matter of high interest, and the level of confidence might vary between imaging modalities. In our study, we assessed the practical confidence of the reading radiologists in ^18^F-FDG PET/MRI and MRI. We found that hybrid PET/MRI imaging has great advantages with regard to the confidence of the final diagnosis that was assigned to a suspicious lesion. We believe that this is before all other reasons based on its ability to visualize pathologically increased glucose metabolism of malignant lesions, thereby, minimizing the uncertainty in the dichotomization between benign and malignant lesion nature compared with conventional imaging techniques [23].

This study has some limitations. First of all, a general limitation of PET/MRI still remains the long acquisition time, reducing the patients comfort during examination [42]. Secondly, since biopsy, especially in patients with advanced tumor stages, was not necessarily required in all lesions according to guideline-based management and ethical standards, a modified reference standard had to be applied including follow-up imaging and clinical examinations. This procedure was in accordance with former studies [15, 19, 28]. Another relative limitation of our study is the fact, that we used the MRI images of the ^18^F-FDG PET/MRI protocol, as some authors prefer a dedicated MRI. However, a comprehensive MRI protocol was established as part of the ^18^F-FDG PET/MRI scan, and, based on this protocol, MRI image quality was not limited compared with a stand-alone MRI.

In conclusion, this prospective study demonstrates a high value of ^18^F-FDG PET/MRI for the N and M staging in patients with primary breast cancer. ^18^F-FDG PET/MRI has a superior diagnostic performance on a per-patient and a per-lesion basis compared with MRI alone when determining the N-stage. Although MRI alone and ^18^F-FDG PET/MRI detected all patients with histopathological proven distant metastases, both modalities bear a certain risk to overestimate the M stage. Nevertheless, ^18^F-FDG PET/MRI should be considered as a useful alternative for systematic staging of breast cancer patients at time of diagnosis.
